# Thyroid function and life expectancy with and without noncommunicable diseases: A population-based study

**DOI:** 10.1371/journal.pmed.1002957

**Published:** 2019-10-25

**Authors:** Arjola Bano, Layal Chaker, Francesco U. S. Mattace-Raso, Natalie Terzikhan, Maryam Kavousi, M. Arfan Ikram, Robin P. Peeters, Oscar H. Franco

**Affiliations:** 1 Department of Internal Medicine and Academic Center for Thyroid Diseases, Erasmus Medical Center, Rotterdam, the Netherlands; 2 Department of Epidemiology, Erasmus Medical Center, Rotterdam, the Netherlands; 3 Institute of Social and Preventive Medicine (ISPM), University of Bern, Bern, Switzerland; 4 Department of Cardiology, Inselspital, Bern University Hospital, University of Bern, Bern, Switzerland; 5 Section of Geriatric Medicine, Erasmus Medical Center, Rotterdam, the Netherlands; Chinese University of Hong Kong, CHINA

## Abstract

**Background:**

Variations in thyroid function within reference ranges are associated with increased risk of diseases and death. However, the impact of thyroid function on life expectancy (LE) with and without noncommunicable diseases (NCDs) remains unknown. We therefore aimed to investigate the association of thyroid function with total LE and LE with and without NCD among euthyroid individuals.

**Methods and findings:**

The study was embedded in the Rotterdam Study, a prospective population-based study carried out in the Netherlands. In total, 7,644 participants without known thyroid disease and with thyroid-stimulating hormone (TSH) and free thyroxine (FT_4_) levels within reference ranges were eligible. NCDs were defined as presence of cardiovascular disease, diabetes mellitus type 2, or cancer. We used the demographic tool of multistate life tables to calculate LE estimates at the age of 50 years, using prevalence, incidence rates, and hazard ratios for three transitions (healthy to NCD, healthy to death, and NCD to death). The total LE and LE with and without NCD among TSH and FT_4_ tertiles were calculated separately in men and women. Analyses were adjusted for sociodemographic and cardiovascular risk factors. The mean (standard deviation) age of the participants was 64.5 (9.7) years, and 52.3% were women. Over a median follow-up of 8 years (interquartile range 2.7–9.9 years), 1,396 incident NCD events and 1,422 deaths occurred. Compared with those in the lowest TSH tertile, men and women in the highest TSH tertile were expected to live 1.5 years (95% confidence interval [CI] 0.8–2.3, *p* < 0.001) and 1.5 years (CI 0.8–2.2, *p* < 0.001) longer, respectively, of which 1.4 years (CI 0.5–2.3, *p* = 0.002) and 1.3 years (CI 0.3–2.1, *p* = 0.004) with NCD. Compared with those in the lowest FT_4_ tertile, the difference in LE for men and women in the highest FT_4_ tertile was −3.7 years (CI −5.1 to −2.2, *p* < 0.001) and −3.3 years (CI −4.7 to −1.9, *p* < 0.001), respectively, of which −1.8 years (CI −3.1 to −0.7, *p* = 0.003) and −2.0 years (CI −3.4 to −0.7, *p* = 0.003) without NCD. A limitation of the study is the observational design. Thus, the possibility of residual confounding cannot be entirely ruled out.

**Conclusions:**

In this study, we found that people with low–normal thyroid function (i.e., highest tertile of TSH and lowest tertile of FT_4_ reference ranges) are expected to live more years with and without NCD than those with high–normal thyroid function (i.e., lowest tertile of TSH and highest tertile of FT_4_ reference ranges). These findings provide support for a re-evaluation of the current reference ranges of thyroid function.

## Abstract 2

Arjola Bano and colleagues reveal that people with a lower-normal level of thyroid function are more likely to live longer and without NCDs.

## Author summary

**Why was this study done?:**

- Thyroid dysfunction is an important public health problem that is associated with an increased risk of noncommunicable diseases (NCDs), such as cardiovascular diseases, diabetes, and cancer. The diagnosis and treatment of thyroid dysfunction is based on thyrotropin and free thyroxine measurements.
- Accumulating evidence has suggested that the clinical consequences of thyroid dysfunction are extended even within the reference ranges of thyrotropin and free thyroxine levels.
- However, the impact of thyroid function on life expectancy with and without NCD remains unknown.

**What did the researchers do and find?:**

- We performed a large prospective population-based cohort study within the framework of the Rotterdam Study.
- We investigated the association of thyroid function with life expectancy with and without NCD among euthyroid individuals.
- We found that individuals with low–normal thyroid function live up to 3.7 years longer overall, of which up to 1.9 years longer with NCD, than individuals with high–normal thyroid function.

**What do these findings mean?:**

- Our study provides novel insights about the qualitative and quantitative impact of thyroid function on life expectancy.
- Our findings provide support for a re-evaluation of the reference ranges of thyroid function in middle-aged and older adults. This can have further implications on the diagnosis and treatment of thyroid disease.

## Introduction

Noncommunicable diseases (NCDs) pose a global health threat, inflicting high disability rates and a huge economic burden [1,2]. According to the World Health Organization, NCDs account for approximately 41 million deaths worldwide each year [2,3]. Global preventive strategies can be beneficial for cardiovascular disease (CVD), diabetes, cancer, and chronic kidney disease (CKD), which account for large proportions of deaths and a large number of years lived with disability [4]. Among other factors, clinical and subclinical thyroid dysfunction have been associated with CVD, diabetes, cancer, and NCD mortality [5–9].

Thyroid dysfunction is defined based on the circulating levels of thyroid-stimulating hormone (TSH) and free thyroxine (FT_4_) [10]. The secretion of these hormones is regulated by the hypothalamus–pituitary–thyroid axis via a negative-feedback mechanism [10]. The reference ranges of TSH and FT_4_ levels are crucial for the diagnosis and treatment of thyroid disease. However, over the past years, there has been an ongoing debate on whether the reference ranges of TSH and FT_4_ levels should be re-evaluated [11,12]. The debate has arisen from studies indicating that not only thyroid disease but also variations in thyroid function within the reference ranges can contribute to the occurrence of chronic conditions and deaths [11,13–15]. Prospective investigations in euthyroid individuals have suggested that high–normal thyroid function increases the risk of CVD, cancer, or CKD, whereas low–normal thyroid function has been associated with an increased risk of chronic metabolic diseases, such as diabetes [11,14–19]. Based on this evidence, it is challenging to determine the balance of overall benefits and risks for specific cutoffs of TSH and FT_4_ levels within the reference ranges [20,21]. In order to capture the pleiotropic effects of thyroid hormones, there is a need for utilizing composite measurements that integrate multiple aspects of health and disease rather than focusing on one adverse outcome alone. A recent study utilizing life expectancy (LE) measurements showed that middle-aged and older adults with low–normal thyroid function have a longer total LE and a longer LE without CVD than those with high–normal thyroid function [22]. Still, it remains unclear whether the differences in LE without CVD reflect healthy years or years lived with other chronic diseases. To address this, multidimensional measures such as LE with and without NCD can provide useful information on the qualitative and quantitative impact of thyroid function on general health.

In a large prospective population-based cohort study, we investigated (1) the association of thyroid function within the reference range with the risk of incident NCD and (2) whether there are differences in the number of years lived with and without NCD, within the reference range of thyroid function. NCDs were defined as the presence of CVD, diabetes, or cancer, which have been highlighted as a global threat by the United Nations and have been prospectively associated with thyroid function [13,14,22,23]. CKD was added to the definition of NCD in a secondary analysis.

## Methods

### Study population

This study was embedded within the Rotterdam Study, a prospective population-based cohort study. The Rotterdam Study investigates the determinants, occurrence, and progression of chronic diseases in middle-aged and older adults from the Netherlands (overall response rate of the study, 72%). The objectives and design have been described in detail previously [24]. The study was initiated in 1989, including 7,983 participants aged 55 years or older. In 2000, the study was extended with a second cohort of 3,011 individuals. In 2006, a third cohort of 3,932 individuals aged 45 years or older was added. Study participants underwent extensive follow-up medical examinations every 3–5 years. Baseline measurements for our study were performed during the third visit of the first cohort (RS-I.3, 1997–1999, *n* = 4,797) and the first visits of the second (RS-II.1, 2000–2001, *n* = 3,011) and third (RS-III.1, 2006–2008, *n* = 3,932) cohorts of the Rotterdam Study. The original cohort during these three visits included a total of 11,740 participants, of which 10,050 had available blood measurements. Thyroid function measurements were performed in a random sample of 9,702 participants. Of these, we excluded 836 participants with past thyroid disease or who were taking thyroid medications, 182 participants without complete information on prevalent or incident NCD, and 1,040 participants with TSH or FT_4_ outside the normal reference ranges. The remaining 7,644 participants with data available on NCD, without known thyroid disease, and with TSH and FT_4_ levels within the reference ranges were eligible. The Rotterdam Study was approved by the Medical Ethics Committee of Erasmus University and by the Ministry of Health, Welfare and Sport of the Netherlands, implementing the Population Study Act Rotterdam Study. In accordance with the Declaration of Helsinki, all participants provided written informed consent. This study is reported per the Strengthening the Reporting of Observational Studies in Epidemiology (STROBE) guideline (S1 STROBE Checklist).

### Assessment of thyroid parameters

Thyroid function was assessed at baseline (RS-I.3 1997–1999; RS-II.1 2000–2001, and RS-III.1 2006–2008) using the same method, assay, and reference ranges. Measurements of TSH and FT_4_ were performed in baseline serum samples stored at −80°C using the Roche electrochemiluminescence immunoassay (ECLIA). The reference ranges of TSH (0.4–4 mIU/L) and FT_4_ (0.86–1.94 ng/dL, alternatively 11–25 pmol/L) were determined based on national guidelines and our previous studies [18,19]. Participants and family physicians were not informed about the results of thyroid function measurements. The study investigators assessing the outcomes of interest were blinded to the thyroid status of participants. Thyroid peroxidase antibodies (TPOAbs) were assessed with the ECLIA for TPOAbs (Roche Diagnostics International, Rotkreuz, Switzerland). TPOAbs > 35 kU/mL were considered positive, as recommended by the assay manufacturer.

### NCD, mortality, and additional measurements

Outcome measures were incident nonfatal NCD, mortality among those with NCD, and overall mortality. NCDs were defined as presence of at least one disease out of CVD, diabetes mellitus type 2, or cancer. All events were independently assessed by two research physicians and further validated by a medical specialist. Information on mortality was obtained from municipality records, general practitioners, and reports of medical specialists [24,25]. CVD was defined as presence of coronary heart disease (CHD), stroke, or heart failure. CHD was defined as coronary revascularization, myocardial infarction, or fatal CHD [25]. Based on the World Health Organization criteria, stroke was defined as a syndrome of rapidly developing symptoms with an apparent vascular cause of focal or global cerebral dysfunction lasting 24 hours or longer or leading to death [26]. Based on the European Society of Cardiology criteria, heart failure was defined as the presence of typical symptoms and signs—breathlessness at rest or during exertion, ankle edema, and pulmonary crepitations—confirmed by the objective evidence of cardiac dysfunction (i.e., chest X-ray, echocardiography) or a positive response to the initiated treatment [27]. Prevalent CVD was assessed at baseline through interview and medical records. After enrollment, participants were monitored for incident CVD through linkage of the study database with files from general practitioners and hospital records. Diabetes mellitus was defined as a fasting serum glucose level ≥ 7 mmol/L, a nonfasting plasma glucose level ≥ 11.1 mmol/L (when fasting samples were absent), or the use of blood glucose–lowering medications [28]. Cases of type 2 diabetes were ascertained at baseline and during follow-up through general practitioners, hospital discharge letters, and serum glucose measurements from the Rotterdam Study visits. Information regarding the use of blood glucose–lowering medications was derived from both structured home interviews and linkage to pharmacy records [28]. Cancer events were classified according to the *International Classification of Diseases* (*ICD*) *10th Edition* [29]. Cases of cancer were determined through general practitioners and hospital discharge letters and by linkage with a nationwide registry of histopathology and cytopathology in the Netherlands, Pathologisch-Anatomisch Landelijk Geautomatiseerd Archief (PALGA) [29]. All types of cancer events were confirmed by pathology records. All cancer events were combined. The most common cancers in the Rotterdam Study are breast cancer, prostate cancer, pancreatic cancer, lung cancer, and colorectal cancer.

### Additional measurements

The baseline home interview provided information on medical history, medication use, tobacco smoking, alcohol consumption, education level, and marital status [24]. Smoking habits were categorized as current, former, and never smoking. Education level was categorized as elementary, lower secondary, higher secondary, and tertiary. Marital status was categorized as single, married, widowed, and divorced/separated. Lipid levels were measured by an automated enzymatic procedure (Mannheim system). Anthropometric measurements were performed in the research center by trained medical staff. Body mass index was calculated as weight in kilograms divided by height in meters squared. Blood pressure was measured in the sitting position on the right arm and calculated as the mean of two measurements using a random-zero sphygmomanometer. In order to perform sensitivity analyses, we also evaluated CKD and chronic obstructive pulmonary disease (COPD). CKD was defined as estimated glomerular filtration rate (eGFR) < 60 ml/min per 1.73 m^2^. eGFR was calculated at baseline and follow-up visits according to the CDK epidemiology collaboration equation [30]. Patients with incident CKD were defined as individuals free of CKD at baseline (eGFR > 60 ml/min per 1.73 m^2^) who had a decline in eGFR to <60 ml/min per 1.73 m^2^ between the two periodic examinations [31]. To estimate the censoring date of the cases, we assumed a linear decrease in eGFR. The date that each case had passed the eGFR threshold of 60 ml/min per 1.73 m^2^ was taken as the censoring date, and it was used to calculate the follow-up time for incident cases. For participants who remained free from CKD, the time spent between the two examinations was used as the follow-up time. COPD was diagnosed based on an obstructive prebronchodilator spirometry (FEV_1_/FVC < 0.70) according to the Global Initiative for Chronic Obstructive Lung Disease (GOLD) guidelines [32]. The incident date of COPD was defined as the date of the first obstructive lung function examination, the date of COPD diagnosis in the medical records, or the date of the first prescription of COPD medications, whichever came first.

### Statistical analyses

An outline of the analysis plan of this study is provided in S1 Text. We did not publish or preregister the analysis plan. The multistate life table is a demographic tool that can estimate the total LE and disease-specific life expectancies using information from people at different ages moving between different health states (e.g., healthy, diseased, dead) [33]. We used multistate lifetables to calculate estimates of total LE and LE with and without NCD among tertiles of TSH and FT_4_ [34]. Multistate life tables combined information from participants in three possible health states—namely, “free of NCD,” “NCD,” and “death.” Possible transitions of participants were (1) from free of NCD to NCD (incident NCD), (2) from free of NCD to death (mortality among those without NCD), and (3) from NCD to death (mortality among those with NCD). Backflows were not allowed, and only the first event into a state was considered [33]. The analyses were performed separately for TSH and FT_4_ tertiles. Because of the known gender differences in LE, analyses were performed separately among men and women. To calculate LE, we followed a similar approach to previous studies [34,35]. We first calculated the prevalence of TSH tertiles among participants with and without NCD, categorized in 10-year age groups. In each transition, we calculated age-specific incidence rates. Next, we applied Poisson regression with Gompertz distribution to compute hazard ratios (HRs) of the association between TSH tertiles and incident NCD or mortality, adjusting for potential confounders. Differences in LE were evaluated using the lowest TSH tertile as a reference. The 95% confidence intervals (CIs) of LE estimates were calculated using the Monte Carlo method with 10,000 bootstrap simulations [36]. We repeated the analyses for the FT_4_ tertiles. Differences in LE were evaluated using the lowest FT_4_ tertile as a reference. Confounders were selected based on biological plausibility and previous literature. Model 1 was adjusted for age, sex, and cohort. Model 2 was adjusted for age, sex, cohort, smoking, alcohol intake, education level, marital status, body mass index, systolic blood pressure, total cholesterol, triglycerides, use of antihypertensive medications, and use of lipid-lowering medications. Multiple imputations were performed in case of missing covariates (<5% for all covariates). Statistical analyses were conducted using IBM SPSS version 21 (IBM), STATA version 13 for Windows (StataCorp), and @RISK software (Palisade).

### Sensitivity analyses

(1) To account for potential reverse causation, we excluded NCD events or deaths that occurred during the first 2 years of follow-up. (2) We excluded participants using thyroid function–altering medications, such as amiodarone or corticosteroids. (3) To detect a potential influence of follow-up duration on our results, we performed the analyses restricting the length of follow-up to 8 years (median follow-up time). (4) CKD was defined by eGFR, measured up to two times within our cohort, and was added to the definition of NCD in a sensitivity analysis. (5) COPD was not included in the primary outcome of NCD, because of the lack of evidence of a pathophysiological association between thyroid function and incident COPD. However, we performed a sensitivity analysis adding COPD to the definition of NCD. (6) We assessed the differences in LE with and without CVD, diabetes, and cancer, separately, among the TSH and FT_4_ tertiles. (7) We calculated unadjusted HRs for incident NCD and death among TSH and FT_4_ tertiles. (8) To account for the potential role of thyroid autoimmunity, we additionally adjusted our analyses for TPOAb positivity. Furthermore, we investigated the association of TPOAb positivity with incident NCD and NCD mortality among those with and without disease.

## Results

A total of 7,644 participants with complete data available on NCD, without known thyroid disease, and with TSH and FT_4_ levels within the reference ranges were considered eligible. Baseline characteristics of eligible participants are presented in Table 1. The mean (standard deviation) age of participants was 64.5 (9.7) years, and 52.3% were women. Over a median follow-up time of 8 years (interquartile range 2.7–9.9 years), 1,396 incident NCD events and 1,422 deaths occurred. Both models yielded similar estimates; therefore, we further report the results of the most adjusted model (model 2), which accounted for age, sex, cohort, smoking, alcohol intake, education level, marital status, body mass index, systolic blood pressure, total cholesterol, triglycerides, use of antihypertensive medications, and use of lipid-lowering medications.

**Table 1:** Baseline characteristics of 7,644 participants.

Baseline characteristics	Men	Women
Number	3,647	3,997
Age, years	64.2 (9.2)	64.8 (10.1)
Smoking, *n* (%)		
Current	897 (24.6)	810 (20.3)
Former	2,143 (58.8)	1,512 (37.8)
Never	607 (16.6)	1,675 (41.9)
Education, *n* (%)		
Elementary	332 (9.1)	588 (14.7)
Lower secondary	1,041 (28.5)	2,012 (50.3)
Higher secondary	1,335 (36.6)	900 (22.5)
Tertiary	939 (25.7)	497 (12.4)
Marital status, *n* (%)		
Single	115 (3.2)	244 (6.1)
Married	3,113 (85.4)	2,474 (61.9)
Widowed	222 (6.1)	870 (21.8)
Divorced/separated	197 (5.4)	409 (10.2)
BMI, kg/m^2^	27.0 (3.5)	27.3 (4.6)
Systolic blood pressure, mm Hg	141.0 (20.3)	137.8 (21.5)
Use of antihypertensive medications, *n* (%)	811 (22.2)	927 (23.2)
Total cholesterol, mmol/l	5.4 (0.9)	5.9 (0.9)
Triglycerides, mmol/l	1.6 (0.9)	1.5 (0.7)
Use of lipid-lowering medications, *n* (%)	662 (18.2)	576 (14.4)
TSH, mIU/L, median (IQR)	1.8 (1.2–2.4)	1.9 (1.3–2.6)
FT_4_, pmol/l	15.9 (2.0)	15.6 (1.9)
TPOAb positive, *n* (%)	187 (5.1)	457 (11.4)

Data are means (sd), unless otherwise specified. TPOAb > 35 kU/mL were considered positive, as recommended by the assay manufacturer.

Abbreviations: BMI, body mass index; FT_4_, free thyroxine; IQR, interquartile range; sd, standard deviation; TPOAb, thyroid peroxidase antibody; TSH, thyroid-stimulating hormone

### Association of thyroid function within the reference range with the risk of NCD and death

The association of TSH tertiles with the risk of incident NCD was not statistically significant (Table 2). Among participants without NCD, the highest TSH tertile was associated with a lower risk of mortality compared with the lowest TSH tertile (HR, 0.67; 95% CI 0.54–0.83; *p*-value < 0.001) (Table 2). Among participants with NCD, the highest TSH tertile had a borderline statistically significant association with a lower risk of mortality than the lowest TSH tertile (HR, 0.88; 95% CI 0.75–1.03; *p*-value = 0.1) (Table 2).

**Table 2:** HRs for incident NCD and death among TSH and FT_4_ tertiles.

			TSH		FT_4_	
Transition	Cases/PY	TSH/FT_4_ tertiles	Model 1 HR (95% CI) (*p*-value)	Model 2 HR (95% CI) (*p*-value)	Model 1 HR (95% CI) (*p*-value)	Model 2 HR (95% CI) (*p*-value)
Incident NCD	1,396/27,705	Tertile 1	1 (Reference)	1 (Reference)	1 (Reference)	1 (Reference)
		Tertile 2	0.98 (0.86–1.11) (0.7)	0.98 (0.86–1.12) (0.8)	1.16 (1.01–1.32) (0.02)	1.17 (1.02–1.33) (0.01)
		Tertile 3	1.05 (0.92–1.19) (0.4)	1.05 (0.92–1.19) (0.4)	1.17 (1.02–1.33) (0.02)	1.17 (1.02–1.34) (0.02)
Mortality among those without NCD	532/32,828	Tertile 1	1 (Reference)	1 (Reference)	1 (Reference)	1 (Reference)
		Tertile 2	0.68 (0.55–0.83) (<0.001)	0.70 (0.56–0.85) (<0.001)	1.21 (0.96–1.51) (0.09)	1.20 (0.95–1.50) (0.1)
		Tertile 3	0.64 (0.52–0.79) (<0.001)	0.67 (0.54–0.83) (<0.001)	1.52 (1.22–1.88) (<0.001)	1.44 (1.15–1.79) (0.001)
Mortality among those with NCD	890/18,456	Tertile 1	1 (Reference)	1 (Reference)	1 (Reference)	1 (Reference)
		Tertile 2	0.90 (0.77–1.06) (0.2)	0.91 (0.78–1.06) (0.2)	1.23 (1.03–1.47) (0.01)	1.24 (1.04–1.47) (0.01)
		Tertile 3	0.84 (0.72–0.99) (0.04)	0.88 (0.75–1.03) (0.1)	1.59 (1.35–1.88) (<0.001)	1.56 (1.32–1.85) (<0.001)

NCDs include cardiovascular disease, diabetes mellitus, and cancer. Poisson regression with Gompertz distribution was used to compute HRs (and 95% CI) for the association of TSH and FT_4_ tertiles with incident NCD and mortality. Model 1: age, sex, and cohort. Model 2: age, sex, cohort, smoking, alcohol intake, education level, marital status, body mass index, systolic blood pressure, total cholesterol, triglycerides, use of antihypertensive medications, and use of lipid-lowering medications.

Abbreviations: CI, confidence interval; FT_4_, free thyroxine; HR, hazard ratio; NCD, noncommunicable disease; PY, person-year; TSH, thyroid-stimulating hormone

The highest FT_4_ tertile was associated with a 1.17-times-higher risk of incident NCD than the lowest FT_4_ tertile (HR, 1.17; 95% CI 1.02–1.34; *p*-value = 0.02) (Table 2). The highest FT_4_ tertile was also associated with a 1.56-times-higher risk of mortality among participants with NCD (HR, 1.56; 95% CI 1.32–1.85; *p-*value < 0.001) and a 1.44-times-higher risk of mortality among participants without NCD (HR, 1.44; 95% CI 1.15–1.79; *p*-value = 0.001) (Table 2), compared with the lowest FT_4_ tertile.

Results for TSH and FT_4_ analyses remained similar in the raw models, after excluding the events that occurred during the first 2 years of follow-up, after excluding users of thyroid function–altering medications, and after additionally adjusting for TPOAb positivity (S1 Table, S2 Table, S3 Table). TPOAb positivity was not associated with the risk of incident NCD, mortality among those without NCD, and mortality among those with NCD (HR, 1.20; 95% CI 0.99–1.44; *p*-value 0.06; HR, 1.24; 95% CI 0.92–1.68, *p*-value = 0.2; HR, 0.97; 95% CI 0.76–1.25; *p*-value = 0.8; respectively).

### Association of thyroid function within the reference range with total LE and LE with and without NCD

**Fig 1:**
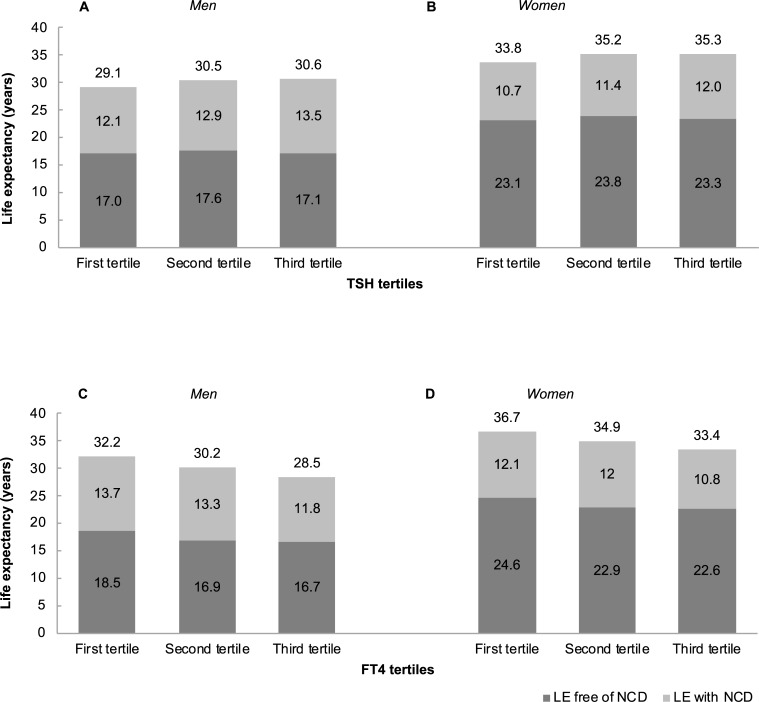
LE with and without NCD at age 50 years among TSH and FT_4_ tertiles in men and women. (A) Estimates of LE with and without NCD (in years) are plotted against TSH tertiles in men. (B) Estimates of LE with and without NCD (in years) are plotted against TSH tertiles in women. (C) Estimates of LE with and without NCD (in years) are plotted against FT_4_ tertiles in men. (D) Estimates of LE with and without NCD (in years) are plotted against FT_4_ tertiles in women. NCDs include cardiovascular disease, diabetes mellitus, and cancer. FT_4_, free thyroxine; LE, life expectancy; NCD, noncommunicable disease; TSH, thyroid-stimulating hormone.

Total LE was higher in the middle than in the lowest TSH tertile and did not change substantially from the middle to the highest TSH tertile (Fig 1). Compared with those in the lowest TSH tertile, men in the highest TSH tertile were expected to live 1.5 years (95% CI 0.8–2.3; *p*-value < 0.001) longer overall; of which 0.1 years (95% CI −0.8 to 1.2; *p*-value = 0.8) longer without NCD and 1.4 years (95% CI 0.5–2.3; *p*-value = 0.002) longer with NCD (Table 3). Compared with those in the lowest TSH tertile, women in the highest TSH tertile were expected to live 1.5 years (95% CI 0.8–2.2; *p*-value < 0.001) longer overall; of which 0.2 years (95% CI −0.8 to 1.3; *p*-value 0.7) longer without NCD, and 1.3 years (95% CI 0.3–2.1; *p*-value 0.004) longer with NCD (Table 3).

**Table 3:** LE with and without NCD at age 50 among TSH and FT_4_ tertiles in men and women.

TSH/FT_4_ tertiles	Differences in total LE (95% CI) (*p*-value)	Differences in LE free of NCD (95% CI) (*p*-value)	Differences in LE with NCD (95% CI) (*p*-value)
**TSH tertiles**^†^			
Men			
Tertile 1	Reference	Reference	Reference
Tertile 2	1.4 (0.5–2.1) (<0.001)	0.6 (**−**0.3 to 1.4) (0.1)	0.8 (**−**0.1 to 1.8) (0.09)
Tertile 3	1.5 (0.8–2.3) (<0.001)	0.1 (**−**0.8 to 1.2) (0.8)	1.4 (0.5–2.3) (0.002)
Women			
Tertile 1	Reference	Reference	Reference
Tertile 2	1.4 (0.6–2.0) (<0.001)	0.7 (**−**0.2 to 1.6) (0.1)	0.7 (**−**0.2 to 1.5) (0.1)
Tertile 3	1.5 (0.8–2.2) (<0.001)	0.2 (**−**0.8 to 1.3) (0.7)	1.3 (0.3–2.1) (0.004)
**FT**_**4**_ **tertiles**^#^			
Men			
Tertile 1	Reference	Reference	Reference
Tertile 2	−2.0 (−3.4 to −0.7) (0.003)	−1.6 (−2.8 to −0.5) (0.006)	**−**0.4 (**−**1.6 to 0.9) (0.5)
Tertile 3	−3.7 (−5.1 to −2.2) (<0.001)	−1.8 (−3.1 to −0.7) (0.003)	−1.9 (−3.4 to −0.3) (0.01)
Women			
Tertile 1	Reference	Reference	Reference
Tertile 2	−1.8 (−3.0 to −0.7) (0.002)	−1.7 (−2.9 to −0.6) (0.003)	**−**0.1 (**−**1.1 to 1.2) (0.8)
Tertile 3	−3.3 (−4.7 to −1.9) (<0.001)	−2.0 (−3.4 to −0.7) (0.003)	**−**1.3 (**−**2.7 to 0.2) (0.07)

NCDs include cardiovascular disease, diabetes mellitus, and cancer. Data are given as years (95% CIs). Multistate life tables were used to calculate LE estimates at the age of 50 years, using prevalence, incidence rates and hazard ratios for three transitions (healthy to NCD, healthy to death, and NCD to death). The 95% CI estimates were calculated using Monte Carlo method. The null hypothesis assumes that there are no differences in LE among tertiles of thyroid function measurements. All life expectancies have been calculated with hazard ratios adjusted for age, cohort, smoking, alcohol intake, education level, marital status, body mass index, systolic blood pressure, total cholesterol, triglycerides, use of antihypertensive medications, and use of lipid-lowering medications.

^†^For this analysis, differences in LE are calculated using the first TSH tertile as reference.

^#^For this analysis, differences in LE are calculated using the first FT_4_ tertile as reference.

Abbreviations: CI, confidence interval; FT_4_, free thyroxine; LE, life expectancy; NCD, noncommunicable disease; TSH, thyroid-stimulating hormone

Total LE decreased progressively with increasing FT_4_ tertiles (Fig 1). Compared with those in the lowest FT_4_ tertile, the difference in LE for men in the highest FT_4_ tertile was **−**3.7 years (95% CI **−**5.1 to **−**2.2; *p*-value < 0.001) overall; of which **−**1.8 years (95% CI **−**3.1 to **−**0.7; *p*-value 0.003) without NCD, and **−**1.9 years (95% CI **−**3.4 to **−**0.3; *p*-value 0.01) with NCD (Table 3). Compared with those in the lowest FT_4_ tertile, the difference in LE for women in the highest FT_4_ tertile was **−**3.3 years (95% CI **−**4.7 to **−**1.9; *p*-value < 0.001) overall; of which **−**2.0 years (95% CI **−**3.4 to **−**0.7; *p*-value 0.003) without NCD and **−**1.3 years (95% CI **−**2.7 to 0.2; *p*-value 0.07) with NCD (Table 3). Results remained similar over the length of follow-up of 8 years (S4 Table) or after including CKD in the definition of NCD (S5 Table) or after including COPD in the definition of NCD (S6 Table). Compared with those in the lowest FT_4_ tertile, men and women in the highest FT_4_ tertile were expected to live fewer years free of CVD, diabetes, and cancer, respectively (S7 Table, S8 Table, S9 Table).

## Discussion

In this large prospective population-based cohort study among middle-aged and older adults, we found differences in LE with and without NCD within the reference ranges of thyroid function. Participants with low–normal FT_4_ levels were expected to live up to 3.7 years longer overall, of which up to 1.9 years longer with NCD than those with high–normal FT_4_ levels. Participants with high–normal TSH levels were expected to live up to 1.5 years longer overall, of which up to 1.4 years longer with NCD than those with low–normal TSH levels. No meaningful sex differences throughout the TSH and FT_4_ tertiles were observed.

Previous studies have suggested that the beneficial effects of high–normal thyroid function on metabolism can be counterbalanced by detrimental effects on other systems, such as the cardiovascular system [13,18,19,22]. In this context, our study sheds light on the resultant system-specific effects of thyroid function, suggesting that the overall risk of NCD increases in the high–normal range of FT_4_ levels. However, the LE estimates not only are attributable to the risk of developing the diseases but also depend on mortality risk. Similar to previous studies conducted in middle-aged and older adults, we showed that high–normal thyroid function is associated with an increased risk of mortality [22,37,38]. This was further translated into a decreased LE with and without NCD for participants with high–normal thyroid function.

More specifically, LE without NCD is the result of two components: risk of incident NCD (transition 1) and risk of mortality among participants without NCD (transition 2). In our study, high–normal FT_4_ levels were associated with a higher risk of incident NCD compared with low–normal FT_4_ levels, leading to earlier clinical manifestation of NCD and fewer years lived without NCD. High–normal FT_4_ levels were also associated with an increased mortality risk among participants without NCD, resulting in a further decrease in total LE and LE without NCD. LE with NCD reflects the combined risk of incident NCD (transition 1) and risk of mortality among participants with NCD (transition 3). We showed that high–normal FT_4_ levels were associated with a 1.17-times-higher risk of incident NCD compared with low–normal FT_4_ levels, meaning an earlier occurrence of NCD across the life span and more years lived with NCD. However, participants with NCD and high–normal FT_4_ levels had an even higher risk of mortality (i.e., 1.56 times higher) than those with low–normal FT_4_ levels, which explains the decrease in the number of years lived with NCD.

Another study from our group previously reported that individuals with low–normal thyroid function were expected to live more years free of CVD than those with high–normal thyroid function [22]. Given the pleiotropic effects of thyroid hormones, the present study provides a broader perspective by using the multidimensional measure of LE with and without NCD, taking into account multiple diseases involving a wide array of mechanisms. Our results indicate that the estimates of LE with and without NCD reflect the combination of all NCDs and are not driven by one chronic disease alone. In a consistent manner, participants with low–normal thyroid function were expected to live more years with CVD, diabetes, and cancer, respectively, than those with high–normal thyroid function. Also, participants with low–normal thyroid function were expected to live more years free of CVD, diabetes, and cancer, respectively, than those with high–normal thyroid function. Together, these differences in the number of years lived with and without diseases contributed to the total differences in LE within the reference ranges of thyroid function.

The added values of this population-based cohort study include the investigation of the association between thyroid function and incident NCD, as well as the evaluation of differences in LE with and without NCD within the reference ranges of thyroid function. Other strengths of the study are the large sample size, the prospective design with a long follow-up period, and the utilization of a multidimensional measure as LE with and without NCD. Participants had extensive and detailed information on covariates including exposures, outcomes, and potential confounders. Events were adjudicated using standardized criteria. Thyroid function measurements were performed before the occurrence of incident NCD events, and event assessors were blinded to the thyroid status of participants. The possibility of reverse causation was taken into account by excluding the events that occurred during the first 2 years of follow-up. Multiple sensitivity analyses provided consistent findings.

Several limitations should also be considered. We did not have data available on triiodothyronine, the active form of thyroid hormone. Nevertheless, TSH and FT_4_ represent the most relevant measurements of thyroid function in clinical practice. We also lacked repeated measurements of thyroid function, even though the normal reference ranges of TSH and FT_4_ levels are shown to be stable over time [39]. In certain conditions (e.g., pregnancy or critical illnesses), the affinity of thyroid hormones to plasma proteins can be affected by substances interfering with the FT_4_ immunoassay. We lacked information on thyroid hormone binding proteins. However, our population consists of community-dwelling middle-aged and elderly people, and therefore, the concentrations of thyroid hormone binding proteins are not expected to have been altered. One can also assume that NCD events may have occurred before the date of diagnosis. To address this, we excluded the events that occurred within the first two years of follow-up; and results remained consistent. Furthermore, the exact date of incident CKD in our study was uncertain because eGFR measurements were performed only twice. We could therefore not include CKD in the primary outcome of NCD. However, we used the repeated measurements of eGFR to determine the slope of eGFR changes over time. Results remained similar after adding CKD to the definition of NCD. Finally, the possibility of residual confounding cannot be entirely ruled out because of the observational character of our study. The Rotterdam Study includes predominantly participants of European descent older than 45 years. Therefore, our findings need to be confirmed in other populations with similar characteristics to our population, as well as in other ethnicities and other age categories.

Our study provides novel insights about the qualitative and quantitative impact of thyroid function on LE, revealing meaningful differences in LE with and without NCD, within the reference range of thyroid function. These results add valuable information to the ongoing discussion on the reference range of thyroid function. Furthermore, these results support a re-evaluation of the reference ranges of thyroid function in middle-aged and older adults, implying the possibility of a downward shift of the FT_4_ current limits. This can, in turn, have further implications on the diagnosis and treatment of thyroid disease.

## Conclusions

In a population of middle-aged and older euthyroid individuals, we found that high–normal FT_4_ levels were associated with an increased risk of incident NCD. Furthermore, we found meaningful differences in LE with and without NCD within the reference ranges of TSH and FT_4_ levels. People with low–normal thyroid function were expected to live more years with and without NCD than those with high–normal thyroid function. These results provide support for a re-evaluation of the current reference ranges of thyroid function. Future studies in other populations are warranted to support the generalization of our findings. Additional research is needed to establish causality and elucidate the underlying mechanisms.

## Supporting information

S1 STROBE checklist STROBE checklist. STROBE, Strengthening the Reporting of Observational Studies in Epidemiology.
(DOCX)

S1 Table Unadjusted HRs for incident NCD and death among TSH and FT_4_ tertiles. FT_4_, free thyroxine; HR, hazard ratio; NCD, noncommunicable disease; TSH, thyroid-stimulating hormone.
(DOCX)

S2 Table HRs for incident NCD and death among TSH and FT_4_ tertiles, additionally adjusted for thyroid peroxidase antibody positivity. FT_4_, free thyroxine; HR, hazard ratio; NCD, noncommunicable disease; TSH, thyroid-stimulating hormone.
(DOCX)

S3 Table HRs for incident NCD and death among TSH and FT_4_ tertiles after excluding the first 2 years of follow-up for NCD and death or after excluding the users of thyroid function–altering medications. FT_4_, free thyroxine; HR, hazard ratio; NCD, noncommunicable disease; TSH, thyroid-stimulating hormone.
(DOCX)

S4 Table LE with and without NCD at age 50 years among TSH and FT_4_ tertiles in men and women over 8 years of follow-up. FT_4_, free thyroxine; LE, life expectancy; NCD, noncommunicable disease; TSH, thyroid-stimulating hormone.
(DOCX)

S5 Table LE with and without NCD at age 50 years among TSH and FT_4_ tertiles in men and women. FT_4_, free thyroxine; LE, life expectancy; NCD, noncommunicable disease; TSH, thyroid-stimulating hormone.
(DOCX)

S6 Table LE with and without NCD at age 50 years among TSH and FT_4_ tertiles in men and women. FT_4_, free thyroxine; LE, life expectancy; NCD, noncommunicable disease; TSH, thyroid-stimulating hormone.
(DOCX)

S7 Table LE with and without CVD at age 50 years among TSH and FT_4_ tertiles in men and women. CVD, cardiovascular disease; FT_4_, free thyroxine; LE, life expectancy; TSH, thyroid-stimulating hormone.
(DOCX)

S8 Table LE with and without diabetes at age 50 years among TSH and FT_4_ tertiles in men and women. FT_4_, free thyroxine; LE, life expectancy; TSH, thyroid-stimulating hormone.
(DOCX)

S9 Table LE with and without cancer at age 50 years among TSH and FT_4_ tertiles in men and women. FT_4_, free thyroxine; LE, life expectancy; TSH, thyroid-stimulating hormone.
(DOCX)

S1 Text Outline of changes made to the analysis plan. (DOCX)

## Abbreviations

CI: confidence interval
CHD: coronary heart disease
CKD: chronic kidney disease
COPD: chronic obstructive pulmonary disease
CVD: cardiovascular disease
ECLIA: electrochemiluminescence immunoassay
eGFR: estimated glomerular filtration rate
FT_4_: free thyroxine
GOLD: Global Initiative for Chronic Obstructive Lung Disease
HR: hazard ratio
*ICD*: *International Classification of Diseases*
LE: life expectancy
NCD: noncommunicable disease
PALGA: Pathologisch-Anatomisch Landelijk Geautomatiseerd Archief
PY: person-year
STROBE: Strengthening the Reporting of Observational Studies in Epidemiology
TPOAb: thyroid peroxidase antibody
TSH: thyroid-stimulating hormone
