# Supplementary material for: Thyroid function and life expectancy with and without noncommunicable diseases: A population-based study
Source: PLoS Med. 2019 Oct 25;16(10):e1002957. doi: 10.1371/journal.pmed.1002957 (PMC6814213; doi:10.1371/journal.pmed.1002957)
Supplement: S1 Table — FT4, free thyroxine; HR, hazard ratio; NCD, noncommunicable disease; TSH, thyroid-stimulating hormone. (DOCX) [file pmed.1002957.s002.docx]

| **Supplemental Table 1. Unadjusted HRs for incident NCD and death among TSH and FT_4_ tertiles** | | | | | | |
| --- | --- | --- | --- | --- | --- | --- |
| **Transition** | **Cases/PY** | **TSH/FT_4_ tertiles** |  | **TSH**  **HR (95% CI) (p-value)** |  | **FT_4_**  **HR (95% CI) (p-value)** |
| Incident NCD | 1396/27705 | Tertile 1 |  | 1 (Reference) |  | 1 (Reference) |
|  |  | Tertile 2 |  | 0.98 (0.86; 1.11) (0.7) |  | **1.19 (1.04; 1.36) (0.01)** |
|  |  | Tertile 3 |  | 1.01 (0.88; 1.14) (0.8) |  | **1.21 (1.07; 1.39) (0.003)** |
|  |  |  |  |  |  |  |
| Mortality among those without NCD | 532/32828 | Tertile 1 |  | 1 (Reference) |  | 1 (Reference) |
|  |  | Tertile 2 |  | **0.68 (0.56; 0.84) (<0.001)** |  | 1.25 (1.00; 1.56) (0.05) |
|  |  | Tertile 3 |  | **0.62 (0.50; 0.76) (<0.001)** |  | **1.60 (1.28; 1.98) (<0.001)** |
|  |  |  |  |  |  |  |
| Mortality among those with NCD | 890/18456 | Tertile 1 |  | 1 (Reference) |  | 1 (Reference) |
|  |  | Tertile 2 |  | 0.91 (0.78; 1.06) (0.2) |  | **1.22 (1.03; 1.46) (0.02)** |
|  |  | Tertile 3 |  | **0.83 (0.71; 0.98) (0.02)** |  | **1.59 (1.34; 1.87) (<0.001)** |
| NCD include cardiovascular disease, diabetes mellitus, and cancer. Poisson regression with Gompertz distribution were used to compute hazard ratios (and 95% CI) for the association of TSH and FT_4_ tertiles with incident NCD and mortality. Abbreviations: HR, hazard ratio; NCD, non-communicable diseases; TSH, thyroid-stimulating hormone; FT_4_, free thyroxine; PY, person-years; CI, confidence interval. | | | | | | |
