# Supplementary material for: Thyroid function and life expectancy with and without noncommunicable diseases: A population-based study
Source: PLoS Med. 2019 Oct 25;16(10):e1002957. doi: 10.1371/journal.pmed.1002957 (PMC6814213; doi:10.1371/journal.pmed.1002957)
Supplement: S3 Table — FT4, free thyroxine; HR, hazard ratio; NCD, noncommunicable disease; TSH, thyroid-stimulating hormone. (DOCX) [file pmed.1002957.s004.docx]

| **Supplemental Table 3. HRs for incident NCD and death among TSH and FT_4_ tertiles, after excluding the first 2 years of follow-up for NCD and death or after excluding the users of thyroid function-altering medications** | | | | | | | | | | |
| --- | --- | --- | --- | --- | --- | --- | --- | --- | --- | --- |
|  |  | |  | **Excluding first 2 years of follow-up for NCD and death** | | |  | **Excluding users of thyroid function-altering medications** | | |
| **Transition** | | **TSH/FT_4_ tertiles** |  | **Cases/PY** | **TSH**  **HR (95% CI) (p-value)** | **FT_4_**  **HR (95%CI) (p-value)** |  | **Cases/PY** | **TSH**  **HR (95% CI) (p-value)** | **FT_4_**  **HR (95%CI) (p-value)** |
| Incident NCD | | Tertile 1 |  | 1036/27312 | 1 (Reference) | 1 (Reference) |  | 1365/27334 | 1 (Reference) | 1 (Reference) |
|  |  | Tertile 2 |  |  | 0.97 (0.83; 1.12) (0.6) | **1.18 (1.01; 1.37) (0.03)** |  |  | 1.00 (0.88; 1.14) (0.9) | **1.16 (1.02; 1.33) (0.02)** |
|  |  | Tertile 3 |  |  | 1.05 (0.90; 1.22) (0.4) | 1.16 (1.00; 1.35) (0.06) |  |  | 1.07 (0.93; 1.22) (0.3) | **1.19 (1.04; 1.35) (0.01)** |
|  | |  |  |  |  |  |  |  |  |  |
| Mortality among those without NCD | | Tertile 1 |  | 385/30677 | 1 (Reference) | 1 (Reference) |  | 515/32341 | 1 (Reference) | 1 (Reference) |
|  |  | Tertile 2 |  |  | **0.74 (0.59; 0.94) (0.01)** | 1.29 (0.99; 1.68) (0.06) |  |  | **0.71 (0.58; 0.87) (0.001)** | 1.16 (0.92; 1.46) (0.2) |
|  |  | Tertile 3 |  |  | **0.60 (0.47; 0.78) (<0.001)** | **1.42 (1.09; 1.84) (0.01)** |  |  | **0.68 (0.55; 0.85) (0.001)** | **1.43 (1.15; 1.79) (0.001)** |
|  |  |  |  |  |  |  |  |  |  |  |
|  | |  |  |  |  |  |  |  |  |  |
| Mortality among those with NCD | | Tertile 1 |  | 682/16599 | 1 (Reference) | 1 (Reference) |  | 849/17991 | 1 (Reference) | 1 (Reference) |
|  |  | Tertile 2 |  |  | 0.94 (0.78; 1.12) (0.5) | **1.27 (1.04; 1.56) (0.02)** |  |  | 0.94 (0.80; 1.10) (0.4) | 1.18 (0.99; 1.41) (0.06) |
|  |  | Tertile 3 |  |  | 0.84 (0.70; 1.02) (0.08) | **1.55 (1.27; 1.87) (<0.001)** |  |  | 0.87 (0.74; 1.03) (0.1) | **1.51 (1.27; 1.79) (<0.001)** |
| NCD include cardiovascular disease, diabetes mellitus, and cancer. Poisson regression with Gompertz distribution were used to compute hazard ratios (and 95% CI) for the association of TSH and FT_4_ tertiles with incident NCD and mortality. Analyses were adjusted for age, sex, cohort, smoking, alcohol intake, education level, marital status, body mass index, systolic blood pressure, total cholesterol, triglycerides, use of antihypertensive medications, and use of lipid-lowering medications. Abbreviations: HR, hazard ratio; TSH, thyroid-stimulating hormone; FT_4_, free thyroxine; NCD, non-communicable diseases; PY, person-years; CI, confidence interval. | | | | | | | | | | |
