# Supplementary material for: Thyroid function and life expectancy with and without noncommunicable diseases: A population-based study
Source: PLoS Med. 2019 Oct 25;16(10):e1002957. doi: 10.1371/journal.pmed.1002957 (PMC6814213; doi:10.1371/journal.pmed.1002957)
Supplement: S4 Table — FT4, free thyroxine; LE, life expectancy; NCD, noncommunicable disease; TSH, thyroid-stimulating hormone. (DOCX) [file pmed.1002957.s005.docx]

| **Supplemental Table 4. LE with and without NCD at age 50 years, among TSH and FT_4_ tertiles, in men and women over 8 years of follow-up** | | | | | |
| --- | --- | --- | --- | --- | --- |
|  | | **Differences in total LE (95% CI) (p-value)** | | **Differences in LE free of NCD (95% CI) (p-value)** | **Differences in LE with NCD (95% CI) (p-value)** |
| **TSH tertiles**† | | | | | |
| *Men* | | | | | |
| Tertile 1 | | Reference | | Reference | Reference |
| Tertile 2 | | **1.8 (1.0; 2.7) (<0.001)** | | 0.3 (-0.7; 1.3) (0.5) | **1.5 (0.5; 2.6) (0.005)** |
| Tertile 3 | | **1.6 (0.6; 2.5) (0.001)** | | 0 (-1.1; 1.0) (1) | **1.6 (0.5; 2.8) (0.006)** |
| *Women* | | | | | |
| Tertile 1 | | Reference | | Reference | Reference |
| Tertile 2 | | **1.7 (1.0; 2.5) (<0.001)** | | 0.5 (-0.6; 1.5) (0.3) | **1.2 (0.2; 2.3) (0.02)** |
| Tertile 3 | | **1.5 (0.7; 2.3) (<0.001)** | | 0.1 (-1.1; 1.2) (0.8) | **1.4 (0.3; 2.6) (0.01)** |
|  |  | |  |  |  |
| **FT_4_ tertiles**# |  | |  |  |  |
| *Men* |  | |  |  |  |
| Tertile 1 | | Reference | | Reference | Reference |
| Tertile 2 | | **-1.5 (-2.9;-0.4) (0.01)** | | **-1.1 (-2.3;-0.1) (0.04)** | -0.4 (-1.8; 0.9) (0.5) |
| Tertile 3 | | **-3.5 (-4.9;-1.8) (<0.001)** | | **-1.7 (-2.9;-0.5) (0.005)** | **-1.8 (-3.3;-0.3) (0.01)** |
| *Women* | | | | | |
| Tertile 1 | | Reference | | Reference | Reference |
| Tertile 2 | | **-1.4 (-2.7;-0.4) (0.01)** | | **-1.2 (-2.6;-0.2) (0.04)** | **-0.2 (-1.4; 1.0) (0.7)** |
| Tertile 3 | | **-3.2 (-4.5;-1.6) (<0.001)** | | **-1.9 (-3.2;-0.6) (0.004)** | **-1.3 (-2.7; 0.2) (0.07)** |
| NCD include cardiovascular disease, diabetes mellitus, and cancer. Data are given as years. Multistate life tables were used to calculate LE estimates at the age of 50 years, using prevalence, incidence rates and hazard ratios for three transitions (healthy to NCD, healthy to death and NCD to death). The 95% CI estimates were calculated using Monte Carlo method. All life expectancies have been calculated with hazard ratios adjusted for age, cohort, smoking, alcohol intake, education level, marital status, diabetes mellitus, body mass index, systolic blood pressure, total cholesterol, triglycerides, use of antihypertensive medications, and use of lipid-lowering medications. †For this analysis, differences in LE are calculated using the first TSH tertile as reference. #For this analysis, differences in LE are calculated using the first FT_4_ tertile as reference. Abbreviations: LE, life expectancy; NCD, non-communicable diseases; TSH, thyroid-stimulating hormone; FT_4_, free thyroxine; CI, confidence interval. | | | | | |
