# Supplementary material for: Thyroid function and life expectancy with and without noncommunicable diseases: A population-based study
Source: PLoS Med. 2019 Oct 25;16(10):e1002957. doi: 10.1371/journal.pmed.1002957 (PMC6814213; doi:10.1371/journal.pmed.1002957)
Supplement: S1 Text — (DOCX) [file pmed.1002957.s011.docx]

**S1 Text.** **Outline of changes made to the analysis plan.**

We did not publish or pre-register a protocol for the analysis of data. As described in the methods section, we followed a clear analysis plan.

a) In view of the ongoing debate on the reference ranges of thyroid function, our study was motivated by the research question “Are there differences in life expectancy (LE) with and without non-communicable diseases (NCD) within the reference ranges of thyroid function?”. As described in the manuscript, we excluded participants without complete information on thyroid function and NCD, participants with thyroid-stimulating hormone (TSH) or free thyroxine (FT_4_) outside the normal reference ranges, and participants with past thyroid disease or taking thyroid medications.

b) The statistical approach was determined before the start of the project and did not change throughout. Multistate lifetables were used to calculate estimates of total LE and LE with and without NCD among tertiles of TSH and FT_4._ Analyses were adjusted for potential confounders, that were selected based on biological plausibility and previous literature. Model 1 was adjusted for age, sex, and cohort. Model 2 was adjusted for age, sex, cohort, smoking, alcohol intake, education level, marital status, body mass index, systolic blood pressure, total cholesterol, triglycerides, use of antihypertensive medications, and use of lipid-lowering medications. Multiple sensitivity analyses were performed.

c) Following feedback from the reviewers, we conducted the following additional sensitivity analyses: (i) We calculated unadjusted hazard ratios for incident NCD and death among TSH and FT_4_ tertiles. (ii) To account for the potential role of thyroid autoimmunity, we additionally adjusted our analyses for thyroid peroxidase antibodies (TPOAb) positivity. Furthermore, we investigated the association of TPOAb positivity with incident NCD events, mortality among those with NCD, and mortality among those without NCD.
